# Guiding the Differentiation Direction of Pancreatic Islet-Derived Stem Cells by Glycated Collagen

**DOI:** 10.1155/2018/6143081

**Published:** 2018-07-03

**Authors:** Gokhan Duruksu, Aysegul Aciksari

**Affiliations:** ^1^Center for Stem Cell and Gene Therapies Research and Practice, Kocaeli University, 41380 Izmit, Kocaeli, Turkey; ^2^Institute of Health Sciences, Stem Cell Department, Kocaeli University, 41380 Izmit, Kocaeli, Turkey

## Abstract

The microenvironment is an important factor of stem cells regulating their maintenance, survival, and differentiation. The glycation of proteins with reducing sugars through nonenzymatic reactions induces the collagen cross-linking, which causes tissue stiffening, which is enhanced during aging and diabetes. In this study, we aimed to analyze the effect of glycated collagen on the stem cell culture and differentiation. The collagen type 1 was modified by glycation with mannose, rhamnose, arabinose, and glucose. After the culture of mesenchymal stem cells on the coated surfaces with glycated collagen, the differences in cell adhesion, proliferation, and differentiation were compared. The results showed that the modifications did not induce apoptosis or cause cell death. However, the culture of cells on modified collagens improved the proliferation. It was found that the mannose-modified collagen stimulated the adipogenic differentiation of stem cells, and rhamnose-modified collagen supports the differentiation into both osteogenic and insulin-producing cells. The low concentration of monosaccharides during glycation process improved the characteristics of the matrix protein in favor of stem cell differentiation. Modification of the collagen by glycation might be used as a tool to improve natural polymers for material-induced stem cell differentiation in the future.

## 1. Introduction

Stem cell differentiation was directed not only by soluble biofactors but also by other factors in the microenvironment of stem cells. The physical aspects, like surface topography [1], stiffness [2], shear stress [3], and light [4], have been shown to guide the differentiation as well. Therefore, surface modification by coating is preferred to control surface roughness and hydrophobicity to stabilize cell attachment and promote cell differentiation [5]. Coating the surface with collagen, laminin, or synthetic polypeptides is the ordinary application in the culture of cells on smooth surfaces, like glass, on which cells loosely bind. In some cases, the coating enables the culture of specific cells, like the feeder-free culture of embryonic stem cells. By designing peptide chains with different length and composition, it was also possible to determine the fate of cell differentiation [6].

In certain circumstances, proteins can also undergo spontaneous modifications in vivo and contribute to age-related diseases. Under the hyperglycolytic conditions, for example, the proteins experience nonenzymatic posttranslational modification leading the formation of advanced glycation end-products (AGEs). Type 1 diabetic patients are especially susceptible to AGE formation. The oxidative condition caused by the accumulation of AGEs in the tissue might lead to biophysical disorders, like Alzheimer, cardiovascular diseases, diabetes, and renal failure [7]. The AGEs, which were formed with age due to the hyperglycemia and hyperlipidemia, are known to change the collagen and other extracellular matrix proteins in tissues [8].

In this study, collagen type 1 was modified by glycation. The effect of this nonenzymatic alteration with four monosaccharides (glucose (G), mannose (M), arabinose (A), and rhamnose (R)) on the cell morphology and the direction of the differentiation was analyzed. The primary aim was to demonstrate the biological effects of the modified collagen by glycation with various monosaccharides on stem cell response and differentiation.

## 2. Material and Methods

### 2.1. Cell Culture

Pancreatic islet-derived mesenchymal stem cells (PI-MSCs) were isolated from rat pancreatic islets by explant and characterized, in the previous study [9]. The cells were maintained in the culture medium (RPMI 1640 (Gibco) supplemented with 10% fetal bovine serum (FBS) and 1% penicillin/streptomycin (Gibco)) at 37°C in 5% CO_2_, humidified atmosphere. The medium was refreshed every two days. The cells were expanded in conventional plastic culture flasks (T75, Corning, Corning, NY, USA). Unless it was mentioned, the cells were seeded on the glass surface for the assays at the density of 3000 cells/cm^2^.

### 2.2. Glycation Collagen

D-(+)-glucose monohydrate, D-(+)-mannose, D-(−)-arabinose, and L-rhamnose monohydrate were supplied from Sigma-Aldrich (Steinheim, Germany). 100 mM monosaccharide solution was prepared in phosphate-buffered saline (PBS) buffer (15 mM, pH 7.4; Gibco, Paisley, UK) separately and mixed with human collagen type I (Cat. number CC050; Millipore, Herts, UK) to 1 mg/ml final solution in PBS buffer. Protein-monosaccharide mixtures were incubated for 14 days at 37°C. Then, collagen solution was dialyzed in Slide-A-Lyzer MINI Dialysis Device (3.5 K MWCO, Thermo Scientific, Waltham, MA, USA) for 16 h against 1000 times the volume of sample with PBS at 4°C.

### 2.3. Surface Coating with Collagen

Glass surfaces were coated with collagen at the concentration of 10 *μ*g/cm^2^ for 24 h at 37°C in 5% CO_2_ humidified atmosphere. The residue solution was removed, and the surfaces were briefly washed with PBS buffer. Without drying the surfaces, cells were cultured. The group of surface coating with unmodified collagen was named “Col,” and the groups coated with glucose-, mannose-, arabinose-, and rhamnose-modified collagen were referred as “Col-G,” “Col-M,” “Col-A,” and “Col-R,” respectively.

### 2.4. Phalloidin Staining

For staining of the actin cytoskeleton with phalloidin-FITC (Sigma-Aldrich), the cells were washed in PBS briefly, fixed with paraformaldehyde (2% in PBS), permeabilized in Triton-X100 (0.1% in PBS) for 60 s and incubated in the phalloidin-FITC solution (1 : 100, in PBS) for 20 min. Following washing twice in PBS, the samples were mounted with mounting medium containing 4′,6-diamidino-2-phenylindole (DAPI; Santa Cruz Biotechnology, Heidelberg, Germany). The cells were investigated under the fluorescent microscope (Leica DMI 4000 B, Wetzlar, Germany).

### 2.5. Cell Proliferation Assay

After culture, the medium was replaced with RPMI 1640 basal medium supplemented with 10% WST-1 reagent (Roche, Mannheim, Germany). After incubating for 2 h, the absorbance at 450 nm was measured by the spectrophotometer (Versa Max microplate reader, Molecular Devices, Sunnyvale, CA, USA), according to the instruction of the manufacturer.

### 2.6. Cell Adhesion Assay

Cell adhesion was evaluated after 16 h of incubation. Attached cells were counted by WST-1 solution (RPMI 1640 basal medium supplemented with 10% WST-1). As a control, cells cultured on the uncoated glass surface were used.

### 2.7. Immunofluorescence Staining

Cells were fixed in methanol (Merck, Darmstadt, Germany) at 4°C for 10 min. After permeabilization with 0.025% Triton X-100 (Merck), the samples were incubated with 1.5% blocking serum (Santa Cruz Biotechnology) in PBS for 30 min at 37°C. After washing three times with PBS, samples were incubated overnight at 4°C with the primary antibody (Supplementary Table 1). After three PBS washes, cells were incubated with secondary antibody (Santa Cruz Biotechnology) for 25 min and mounted with mounting medium containing DAPI (Santa Cruz Biotechnology).

### 2.8. LDH Toxicity Assay

Cytotoxicity was determined by lactate dehydrogenase (LDH) assay with Cytotoxicity Detection Kit LDH (Roche), according to the manufacturer's instructions. The cell culture on the uncoated glass surface was used as a control.

### 2.9. Gene Expression Analysis

Total RNA was extracted with High Pure RNA Isolation Kit (Roche), according to the manufacturer's instructions. After synthesis of single strand cDNA by Transcriptor High Fidelity cDNA Synthesis Kit (Roche), gene expressions were carried out by LC480 DNA SYBR Green I Master (Roche) with gene-specific primers (Supplementary Table 2) on LightCycler 480-II instrument (Roche), according to the manufacturer's protocol. Data were analyzed with the LC480 SW1.5 software.

### 2.10. Osteogenic Differentiation

Osteogenic differentiation was induced by RPMI 1640 medium supplemented with 0.1 *μ*M dexamethasone (Sigma-Aldrich), 0.05 mM ascorbate-2-phosphate (Sigma-Aldrich), 10 mM *β*-glycerophosphate (Sigma-Aldrich), 1% penicillin/streptomycin (Gibco), and 10% FBS. The medium was refreshed twice a week. At the end, the differentiation was estimated by Alizarin Red staining. For Alizarin Red staining, cells were fixed for 5 min in 70% ethanol. The cells were stained with Alizarin Red solution (2%, pH 4.2). Stained cells were dehydrated in pure acetone, fixed in acetone-xylene (1 : 1) solution, and cleared with xylene. Differentiation on the uncoated glass surface was defined as the control group. The differentiated cells were analyzed by Western blotting with osteocalcin antibody (Supplementary Table 1).

### 2.11. Adipogenic Differentiation

The adipogenic differentiation was induced by Mesencult MSC Basal Medium supplemented with 10% adipogenic differentiation supplement (Stemcell Technologies, Vancouver, BC, Canada) and 1% penicillin/streptomycin for 3 weeks. The medium was refreshed every 2–4 days. Intracellular lipid droplets indicating adipogenic differentiation, which was analyzed by Oil Red O staining (0.5%; Sigma-Aldrich) and confirmed by Western blotting using the antibody for adiponectin (Supplementary Table 1). Differentiation on the uncoated glass surface was defined as the control group.

### 2.12. Endocrine Cell Differentiation

Cells were induced by DMEM/F12 basal medium (Gibco) supplemented with 10% FBS, 1% penicillin/streptomycin, 17.5 mM glucose, 25 ng/ml hEGF (Biological Industries, Kibbutz Beit Haemek, Israel), 10 mM nicotinamide (Sigma-Aldrich), 2 nM activin-A (Sigma-Aldrich), 10 nM exendin-4 (Sigma-Aldrich), 100 pM hHGF (Biological Industries), and 10 nM pentagastrin (Sigma-Aldrich). Differentiation was assessed by gene expression, Western blotting, and immune staining (Supplementary Table 1). Differentiation on the uncoated glass surface was defined as the control group.

### 2.13. Western Blotting Analysis

After the differentiation of the cells on the coated surfaces, the cells were lysed by Mammalian Protein Extraction Reagent (M-per, Thermo Scientific, Rockford, IL, USA) on the culture flask, centrifuged at 14,000*g* for 5 min at room temperature and the supernatant was collected. The total protein concentration was determined by BCA assay. For Western blotting, 6 *μ*g of protein sample was mixed with the dye solution (Bolt LDS Sample Buffer, Thermo Scientific, Carlsbad, CA, USA) and the reducing agent (Bolt Sample Reducing Agent), according to the instructions provided by the supplier. The protein mix was centrifuged and denatured at 70°C for 10 min. Then, the mixture was loaded on ready-to-use 4–12% Bis-Tris Plus mini-gels (Thermo Scientific). The gels were run at 200 V constant for 30 min using MES-SDS running buffer (Thermo Scientific). The iBlot2 dry blotting system was used to blot the proteins on the nitrocellulose membrane. iBind western processing device (Thermo Scientific) was used to stain the membrane. After incubation of the membrane with primary and secondary antibodies (Supplementary Table 1), the detection was performed with LumiGLO Reagent (Cell Signaling Technology, Danvers, MA, USA) by MF-ChemiBIS3.2 (DNR Bioimaging Systems, Jerusalem, Israel), according to the protocol's instruction.

### 2.14. Statistical Analysis

All experiments were repeated at least three times. Data were analyzed using unpaired Student's *t*-test in conjunction with the Newman–Keuls test and analysis of variance for repeated measures where appropriate. The significance of the results was calculated by SPSS 10.0 (SPSS Inc., Chicago, IL, USA). Differences between the experimental and control groups were regarded as statistically significant when *p* < 0.05.

## 3. Results

### 3.1. Effect of Modified Collagens on Cell Morphology

**Figure 1:**
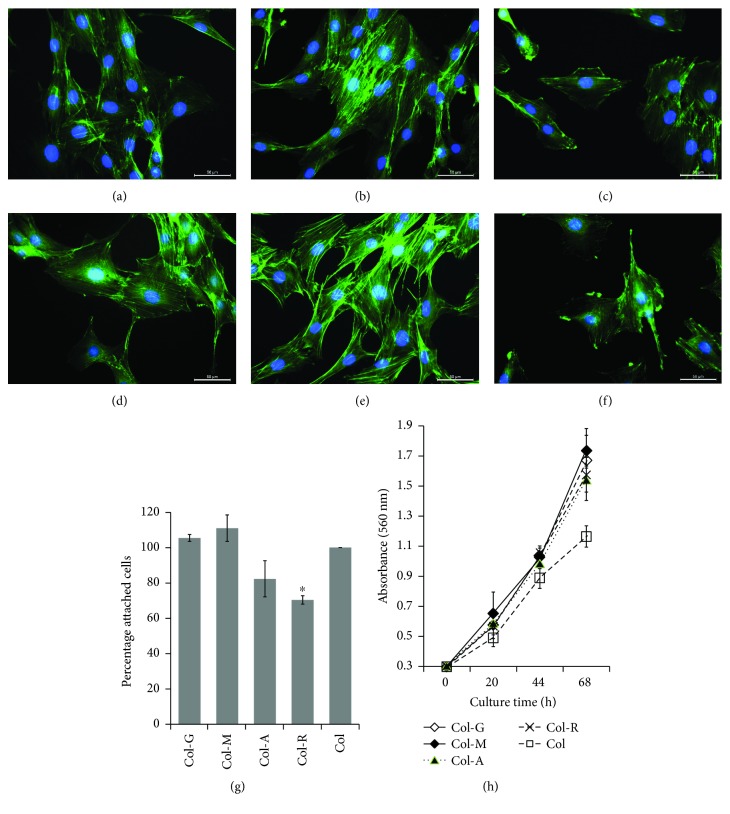
The cytoskeleton structure of PI-MSCs cultured on Col (b), Col-G (c), Col-M (d), Col-A (e), and Col-R (f) was shown after the staining with FITC-phalloidin (scale bars: 50 *μ*m). Uncoated glass surface (a) was used as a control. The cell attachment on the collagen surfaces was given as the percentage with respect to the unmodified Col (g). Only cells on the rhamnose-modified collagen showed a significant decrease in the cell attachment (^∗^ *p* < 0.05). The alteration of the proliferation rates after the culture on the collagens was estimated by WST-1 assay (h). All the surfaces coated with modified collagens supported the cell proliferation higher than the unmodified collagen (*p* < 0.05).

The effect of modified collagen on cell morphology and proliferation was analyzed by F-actin staining with phalloidin and WST-1 assay, respectively (Figure 1). The phalloidin staining showed the differences in cytoskeleton assembly in PI-MSCs in response to the surface coating, and the parallel fibers stretched throughout the cytoplasm showed the intactness of the cells. Despite the cells cultured on glass surface had weak staining, the cells on collagen (Figure 1(b)) showed the formation of dense thick actin fibers. The staining was even stronger in the cells on Col-A compared to the cells on Col (Figures 1(b) and 1(e)). The culture on glucose-, mannose-, and rhamnose-modified collagens reversed the supportive effect of unmodified collagen; the intercellular actin structures preserved but thin short fibers were visible (Figures 1(c), 1(d), and 1(f)). The actin structure highly degenerated after culture on Col-R, and the cells appeared loosely attached. The cell adhesion assay showed the weak interaction of cells to the surface in Col-R group compared to Col group (Figure 1(g)). The cell adhesion was slightly improved in the cells on glucose and mannose modified collagens, but the difference was not statistically significant compared to unmodified collagen. The cell proliferation considerably improved by culturing on the modified collagens (Figure 1(h)). The proliferation rate increased twice, and the number of cells became statistically significant after 68 h compared to the culture on unmodified collagen.

### 3.2. Evaluation of Apoptosis in the Cells

**Figure 2:**
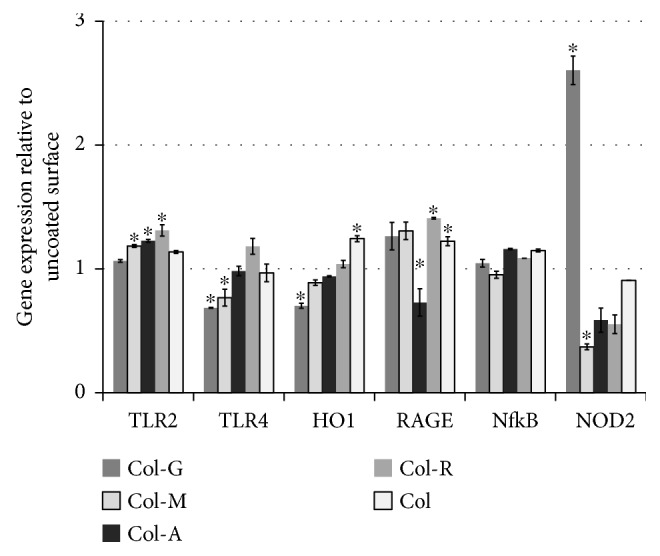
Expression of proinflammatory genes in PI-MSCs cultured on the glycated collagens. The cells were cultured for 72 h, and the expression of toll-like receptor 2 (TLR2), TLR4, NF-*κ*B, heme oxygenase-1 (HO1), receptor for advanced glycation end-products (RAGE), and nucleotide oligomerization domain 2 (NOD2) were estimated. Gapdh served as the housekeeping gene, and data were calculated using the 2^−ΔΔCt^ method. The significant difference compared to the cells cultured on the unmodified collagen group was shown by ^∗^ *p* < 0.05.

Both the expression of apoptotic genes and the active Caspase 3 staining were used to evaluate the apoptosis in the PI-MSCs cultured on the modified collagens (Figure 2), but no staining was observed for active Caspase 3 in any group (data not shown). Furthermore, LDH activity was also insignificant for the modified collagens groups, which showed less than 5% toxicity.

Toll-like receptor expression provoked by AGEs was analyzed for any inflammatory response. Although TLR2 expression was slightly increased in cells on Col-R, this change was not significant compared to cells on unmodified collagen. The change in the TLR4 gene expression was more significant than TLR2. TLR4 expression was decreased 40% in Col-G and 25% in Col-M compared to Col. On the contrary, Col-R group demonstrated slightly increased expression of TLR4. The oxidative stress marker, HO1, increased in the cells cultured on Col, while the expression was low on Col-G. NF-*κ*B expressions were not changed significantly in the groups of glycated collagens. The expression of RAGE was slightly increased in all groups except for the cells on Col-A (Figure 2). The expression of NOD2 has significantly induced in the cells cultured on glucose-modified collagen about 2.5 times of control. Mannose-, rhamnose-, and arabinose-modified collagens did not induce RAGE expression. The culture on Col-A and Col-R repressed the expression of RAGE twice and on Col-M about three times (Figure 2).

### 3.3. The Effect of Collagens on the Adipogenic and Osteogenic Differentiation

**Figure 3:**
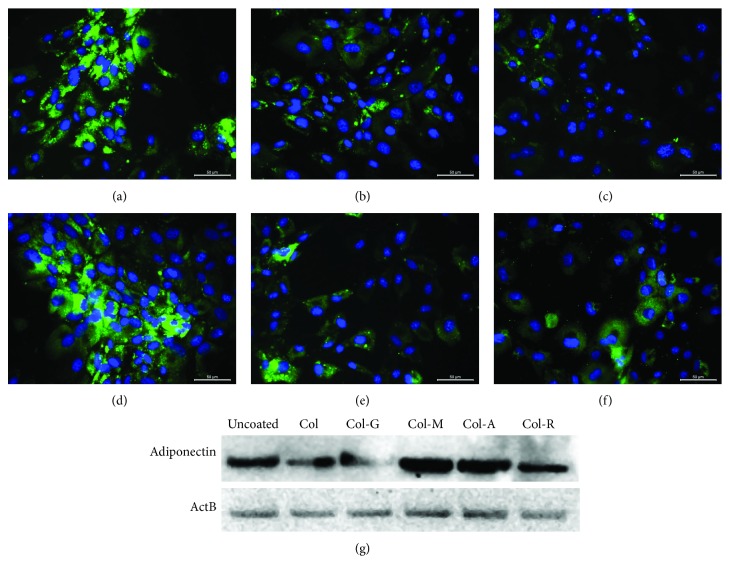
Adipogenic differentiation of PI-MSCs on modified collagen. The effect of the glycated collagens on differentiation was compared with cells differentiated on the uncoated glass surface (a). The stem cells were seeded on the glass surface coated with unmodified Col (b), Col-G (c), Col-M (d), Col-A (e), and Col-R (f) and differentiated for 22 days. The adipogenic differentiation was assessed by immunohistochemical staining for adiponectin (green; a–f) and Western blot analysis for adiponectin (g). The nuclei were stained with DAPI (blue). Scale bar: 100 *μ*m.

PI-MSCs on the uncoated glass surface successfully differentiated into adipocytes (Figure 3(a)). However, the adiponectin expression level decreased in the cells on Col, Col-R, and Col-A (Figures 3(b), 3(e), and 3(f)). The differentiation of cells on Col-G suppressed significantly too (Figure 3(c)). However, adiponectin expression strongly induced in the cells differentiated on Col-M (Figure 3(d)). Col-M and uncoated glass surface groups showed similar staining intensity of adiponectin, but the main difference was observed in the number of oil droplets, which was observed clearly only in the cells on the uncoated surface.

**Figure 4:**
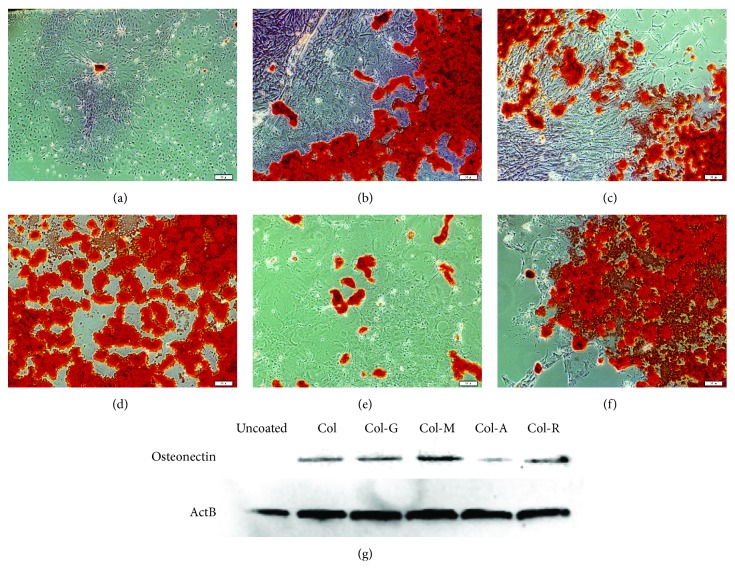
Osteogenic differentiation of PI-MSCs on modified collagen. The stem cells were seeded on the glass surface coated with Col (b), Col-G (c), Col-M (d), Col-A (e), and Col-R (f) and differentiated for 24 days. The calcium deposits (red) was analyzed by Alizarin Red S staining (a–f), and the differentiation was confirmed by Western blot analysis for osteocalcin (g). The effect of glycated collagens on differentiation was determined by comparing with the cells differentiated on the uncoated glass surface (a). Scale bar: 100 *μ*m.

Unlike adipogenic differentiation, cells on uncoated glass surface showed poor osteogenic differentiation capacity (Figure 4(a)). However, the osteogenic differentiation efficiency was higher in cells on Col, Col-M, and Col-R (Figures 4(b), 4(d), and 4(f)). Although osteogenic differentiation capacity decreased in the cells on Col-G, the most significant effect was observed in the cells differentiated on Col-A, which showed poor staining by Alizarin Red S. In Col-A, osteogenic differentiation found to be as weak as in the cells on the uncoated glass surface (Figure 4(e)).

### 3.4. Differentiation into Endocrine Cell Lineages

**Figure 5:**
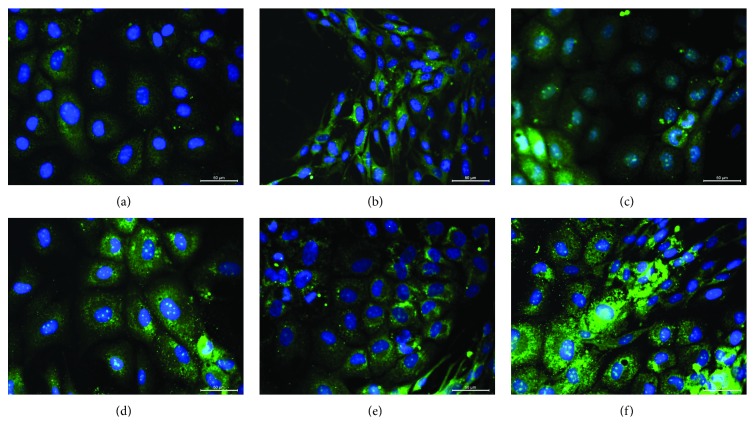
Endocrine differentiation of stem cells on modified collagens. After 28 days of differentiation, the differentiation was assessed by immune staining against insulin. With respect to the cells on the uncoated (a) and collagen coated (b; unmodified) surfaces, the differentiation was more efficient in the cells cultured on the Col-M (d), Col-A (e), and Col-R (f). However, Col-G (c) suppressed the endocrine differentiation in PI-MSCs. Scale bar: 50 *μ*m.

The differentiation efficiency into beta-like cells was low in the cells differentiated on the uncoated glass surface (Figure 5(a)). On Col, the differentiation potential of PI-MSCs improved, as it was in the osteogenic differentiation. The remarkable change during the differentiation in this group was in the nucleus size, which appeared smaller in Col group than the control group.

**Figure 6:**
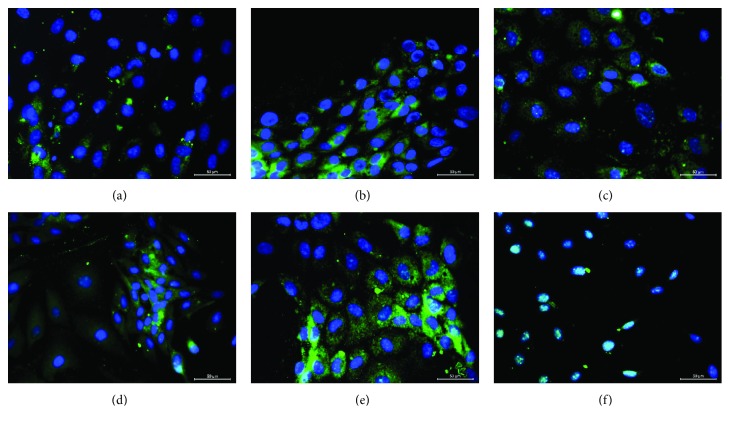
PDX1 staining of PI-MSC differentiated into endocrine cell lineage on modified collagens. The staining was observed in the cells differentiated on Col-G (c), Col-M (d), and Col-R (f), but the expression was higher on Col (b) and Col-A (e). Unlike the others, PDX1 expression in cells on Col-R-coated surface was in the nucleus. Scale bar: 50 *μ*m.

The highest insulin expressions were observed in the cells differentiated on Col-M and Col-R. The presence of transcription factor PDX1 in those cells was shown to confirm the insulin expression. PDX1 is an important marker in the respect of being both a differentiation indicator and a regulatory factor for insulin production. According to the immune fluorescence staining against PDX1, its level was low in the cells differentiated on the uncoated and Col-G coated glass surfaces (Figures 6(a) and 6(c)). The moderate expression was observed in PI-MSCs on Col and Col-M (Figures 6(b) and 6(d)). The highest staining was observed in the cells on Col-A (Figure 6(e)). Despite the promising results, all the staining patterns were cytoplasmic except for the PI-MSCs differentiated on Col-R, in which the PDX1 staining pattern was nucleic (Figure 6(f)).

**Figure 7:**
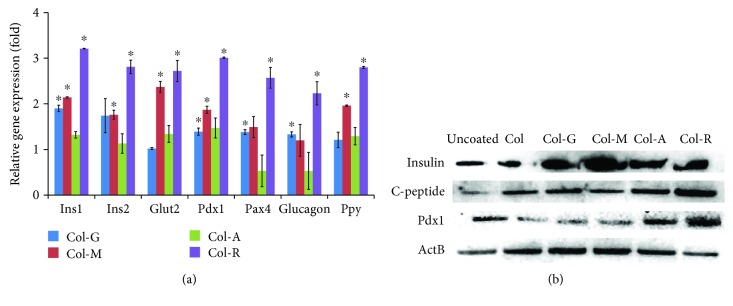
Comparison of gene expressions of pancreatic islet cell (alpha, beta, and PP-cells) markers in the differentiated PI-MSCs on modified collagen surfaces (a). Rhamnose-modified collagen substantially supported the beta cell differentiation (Ins1/2, Glut2, Pdx1, and Pax4) but also PP-cell (Ppy) and alpha cell (glucagon) differentiation. The Western blot analysis (b) for C-peptide and Pdx1 also verified the effect of rhamnose-modified collagen on beta cell differentiation. The significant gene expression level compared to the unmodified collagen group was shown by ^∗^ *p* < 0.05.

The expression of alpha, beta, and gamma (PP) cell markers in PI-MSCs was estimated relative to the expression in the cells differentiated on Col (Figure 7). The cells on G-Col only showed slightly increased insulin expression, but the other markers were not significantly changed. After the differentiation by chemical induction, the marker gene expressions in PI-MSCs on Col-A did not alter significantly, but only Pax4 and Glucagon (alpha cell marker) expressions were reduced twice. The endocrine cell differentiation on Col-M improved the efficiency, but the improvement was observed highest in the PI-MSCs on Col-R. Ins1/2, Pdx1, and PPy (pancreatic polypeptide) were expressed twofold higher, and most significantly the expression of Glut2 increased to 2.5 times of the expressions in the cells differentiated on Col. All the expression levels of endocrine differentiation markers were increased in the cells differentiated on Col-R. The noticeable response was observed in Ins1, Pdx1, and PPy genes with a threefold increase in the expression.

## 4. Discussion

Collagen was modified by reducing monosaccharides in this study to imitate the changes, which spontaneously take place with aging and cause age-related atrophic changes in tissues. To observe the diverse effect of modifications, collagen was treated with different types of monosaccharides beside the glucose. The actin structures of cells cultured on modified collagens showed distinct variations in the organization depend on the type of monosaccharide. While mannose- and arabinose-conjugated collagens supported the intactness of the actin structures in PI-MSCs during the culture, F-actins became tenuous, thinner, and abnormally distributed in Col-G and Col-R groups, but neither decrease in cell proliferation nor induction of apoptosis was observed in any group. Despite the number of attached cells on Col-R was significantly low, it did not affect the stability of the cell culture. Although other studies indicated that AGEs inhibited the proliferation of MSCs and reduced the cell numbers [10], this was not observed during the culture. It could be explained by dose-dependent effect for AGEs [11]. In our study, the coating process was performed with 10 *μ*g/ml collagen while the concentration of AGE-products varied between 100 and 400 *μ*g/ml in other studies. At low concentrations, the proliferation of mesangial cells was supported whereas it was inhibited at high AGE concentrations [12, 13]. Similarly, the proliferation rate in groups with modified collagens was improved significantly compared to the control in our study.

The apoptosis was not observed in the cell culture. TLR-mediated signaling induced by glycated products was reported responsible for apoptosis signaling, and the expression of RAGE and NF-*κ*B was induced by TLR2 and TLR4 [14]. However, their protective effects in ischemia-reperfusion injury were demonstrated, as well [15]. The weak activation of TLR signaling in PI-MSCs was demonstrated to modulate their cellular function and therapeutic effects [16]. TLRs are responsible for the recognition of pathogen-associated molecular patterns, and, therefore, they are associated with the primary immune response against the pathogens, TLR2 senses peptidoglycan and TLR4 lipopolysaccharide [17]. Similarly, the glycated collagens might also interact with those receptors, which were found to be also expressed by MSCs [18]. Beside the protection against infections, TLR might also involve in the differentiation character of MSCs, such that TLR2 and TLR4 activation might promote osteogenic differentiation [19] without affecting their adipogenic differentiation potential [20]. However, the response of MSCs to TLR induction alters with the cell source. The TLR2 might both induce osteogenic differentiation and repress adipogenic differentiation at the same time [17]. TLR2 and TLR4 expressions were upregulated in the cells cultured on Col-R significantly, which might be the reason for the rather higher osteogenic differentiation efficiency compared to the other groups. However, the cells cultured on Col-M showed lower TLR4 expression while they differentiated efficiency into both osteogenic and adipogenic cell lineages in the distinct experimental setup. This controversial result might be explained by the heterogeneous nature of the glycated collagen.

The expression of RAGE in human MSCs was demonstrated to cause loss of their differentiation ability [10]. TLR and NOD-mediated signaling were reported to promote osteogenic differentiation of MSCs while suppressing adipogenic differentiation [21]. In our study, the cells cultured on Col-G showed low TLR4 and high NOD2 expressions that might affect their commitment to differentiate into osteogenic cells and suppression of adipogenic differentiation. On the contrary, NOD2 expression was downregulated most significantly in the cells cultured on Col-M but also on Col-A and Col-R. This downregulation might also induce the efficiency of adipogenic differentiation in PI-MSCs on Col-M. Interestingly, the Col-R surface induced both osteogenic and adipogenic differentiation. It is unclear whether the decrease in cell adhesion might affect the RAGE expression, but it might influence the activity of signaling pathways regulating the differentiation [22]. Both NOD2 and TLR expressions might influence osteogenic differentiation, but only NOD2 could inhibit the adipogenic differentiation while TLRs had no effect [21]. The low-level expression of NOD2 might induce adipogenic differentiation, and at the same time, the induction of TLR2/4 supported osteogenic differentiation. The arabinose-modified collagen affected differently on the osteogenic differentiation capacity compared to others. In this group, the cells showed decreased cell adhesion. As the structural integrity was reported to be required for osteogenic differentiation [23, 24], decreased actin polymerization might suppress osteogenic differentiation.

In the endocrine cell differentiation medium, PI-MSCs expressed insulin and Pdx1 at varying levels depending on the coated surface on which they were cultured. In the previous study with PI-MSCs, they were shown to differentiate into insulin-producing cells in the defined chemical cocktail [9, 25]. By using modified collagens in this study, the differentiation was supported further. The highest expression of beta cell markers was observed in Col-R group, but the genes of other pancreatic islet cells, like glucagon-secreting alpha cells, were also expressed at the same time. Although the immune staining of PDX1 was stronger in Col-A group, the staining pattern was more specific in PI-MSCs on Col-R, in which immune reactivity was observed in the nucleus. This distinct staining in the nucleus might indicate glucose-dependent insulin production of cells on Col-R. The cell adhesion was decreased in the cells cultured on Col-R, but the generation of functional beta-like cells was supported.

The studies related to the RAGE were shown that the maintenance mechanism of BM-MSCs could be disrupted during the stimulation of RAGE signaling [26]. The RAGE knockout MSCs demonstrated better differentiation capacity into adipocytes and osteocytes. In diabetes, the RAGE signaling becomes chronic and the maintenance progress of MSCs might be reduced. In the Col-A group, the MSCs showed significantly lower RAGE expression compared to the other groups. However, the differentiation capacity became worse than the unmodified collagen group cells instead of the improvement. The deleterious effect of glycated protein on MSCs might be its proinflammatory characteristics. In our findings, the induction of inflammation was insignificant, which might be due to the decoy effect of the soluble RAGE (sRAGE) present in the culture medium [27]. These proteolytically truncated forms of RAGE and their high level in the serum of type 2 diabetes were also reported to induce the expression of inflammatory markers in other studies as well [26]. In the present study, the isoforms of sRAGE might be formed and involved into cellular processes. However, the effect of soluble RAGE on MSC differentiation was analyzed, and no effect was found on the differentiation capacities of these cells into osteo-, adipo-, or chondrogenic cell lines.

The modification of collagen by glycation at low reducing sugar concentrations made it possible to improve the characteristics of the matrix polymer in favor of stem cell differentiation. By the modification of natural polymers, it could be possible to provide new features to the matrix proteins. Thereby, it might be possible to control the differentiation direction of the stem cells and to improve in generating functional mature cells. The modification of collagen by glycation is an irreversible process and has the chronic effect on tissue function and maintenance. It was found that the glycated collagen with glucose might have a deleterious effect, but the other reducible sugar acted differently, which should be analyzed in details.

## Data Availability

The data used to support the findings of this study are available from the corresponding author upon request.

## Supplementary Materials

Supplementary 1 Supplementary Table 1: sequence of gene-specific primers used in real-time PCR.

Supplementary 2 Supplementary Table 2: primary antibodies used in immunofluorescence staining.
