# Supplementary material for: Guiding the Differentiation Direction of Pancreatic Islet-Derived Stem Cells by Glycated Collagen
Source: Stem Cells Int. 2018 Jul 3;2018:6143081. doi: 10.1155/2018/6143081 (PMC6051021; doi:10.1155/2018/6143081)
Supplement: Supplementary 1 — Supplementary Table 1: sequence of gene-specific primers used in real-time PCR. [file 6143081.f1.pdf]

Supplementary Table 1. Primary Antibodies Used in Immunofluorescence (IF) Staining and Western Blot (WB) Analysis

**Antibody**	**Supplier**	**Cat. No.**	**Application**
Active Caspase3	Millipore	AB3623	IF
Adiponectin (ACRP30)	Santa Cruz Biotechnology	sc-26497	IF and WB
C-peptide (proinsulin)	Santa Cruz Biotechnology	sc-52033	WB
Insulin	Santa Cruz Biotechnology	sc-9168	IF and WB
Osteocalcin	Santa Cruz Biotechnology	sc-30044	WB
Pdx1	Santa Cruz Biotechnology	sc-14664	IF and WB
Actin beta	Santa Cruz Biotechnology	sc-8432	WB
