# Supplementary material for: Guiding the Differentiation Direction of Pancreatic Islet-Derived Stem Cells by Glycated Collagen
Source: Stem Cells Int. 2018 Jul 3;2018:6143081. doi: 10.1155/2018/6143081 (PMC6051021; doi:10.1155/2018/6143081)
Supplement: Supplementary 2 — Supplementary Table 2: primary antibodies used in immunofluorescence staining. [file 6143081.f2.pdf]

Supplementary Table 2. Sequence of Gene-Specific Primers Used in Real-Time PCR

**Gene (Acc. No.)**	**Full Name**	**Forward Primer**	**Reverse Primer**
TLR2 (NM_198769.2)	toll-like receptor 2	GTACGCAGTGAGTGGTGCAAGT	GGCCGCGTCATTGTTCTC
TLR4 (NM_019178.1)	toll-like receptor 4	AATCCCTGCATAGAGGTACTTCCTAAT	CTCAGATCTAGGTTCTTGGTTGAATAAG
NF-κB (NM_001276711.1)	nuclear factor kappa B	AGAGAAGCACAGATACCACTAAG	CAGCCTCATAGAAGCCATCC
HO1 (NM_012580.2)	heme oxygenase-1	GGTGTCCAGGGAAGGCTTTAAG	GTGCAGCTCCTCAGGGAAGTAG
RAGE (NM_053336.2)	receptor for advanced glycation end-products	CTACCTATTCCTGCAGCTTC	CTGATGTTGACAGGAGGGCTTTCC
NOD2 (NM_001106172.1)	nucleotide oligomerization domain 2	TTCTGCCTTACGAGGGTTACTCTCT	ATGGTCCTCAGCTTAGCAGTGAAC
Ins1 (NM_019129.3)	insulin 1	GACCTTGGCACTGGAGGTT	CCAGTTGGTAGAGGGAGCAG
Ins2 (NM_019130.2)	insulin 2	CGAAGTGGAGGACCCACA	TGCTGGTGCAGCACTGAT
Pdx1 (NM_022852.3)	pancreatic and duodenal homeobox 1	CGTAGTAGCGGGACAACGA	CTCCTCGCCCGAGGTTAC
Glut2 (NM_012879.2)	glucose transporter type 2	TAGAGCAGCTCTTTATTCCAGATTT	TTACTCTCCATTTCAGTCCTTTGT
Pax4 (NM_031799.1)	paired box 4	GAAGCTTATGCAGCAGGACGGTCTC	CGGATCCTTATGGCCAGTGTAAGTAATAG
Gcg (NM_012707.2)	glucagon	GGTGAAAGGCCGAGGAAG	GAGAAGGATCCATCAGCATGT
PPy (NM_012626.2)	pancreatic polypeptide	ACTCGCTCAGGACACAGGAT	CGGGTACATTGGCTCCAG
Gapdh (NM_017008.4)	glyceraldehyde-3-phosphate dehydrogenase	AGAGAGAGGCCCTCAGTTGCT	TGGAATTGTGAGGGAGATGCT
